# Phosphorylation and functionality of CdtR in *Clostridium difficile*

**DOI:** 10.1016/j.anaerobe.2019.102074

**Published:** 2019-08

**Authors:** T.W. Bilverstone, N.P. Minton, S.A. Kuehne

**Affiliations:** a Clostridia Research Group, BBSRC/EPSRC Synthetic Biology Research Centre (SBRC), School of Life Sciences, Centre for Biomolecular Sciences, The University of Nottingham, Nottingham, NG7 2RD, UK; b NIHR Nottingham Biomedical Research Centre, Nottingham University Hospitals NHS Trust and the University of Nottingham, Nottingham, NG7 2RD, UK; c Oral Microbiology Group, School of Dentistry and Institute of Microbiology and Infection, College of Medical and Dental Sciences, The University of Birmingham, Birmingham, B5 7EG, UK

**Keywords:** *C. difficile*, CDT, CdtR, Binary toxin, Virulence, Two-component system

## Abstract

The production of TcdA, TcdB and CDT in *Clostridium difficile* PCR ribotype 027, is regulated by the two-component system response regulator CdtR. Despite this, little is known about the signal transduction pathway leading to the activation of CdtR. In this study, we generated R20291ΔPalocΔ*cdtR* model strains expressing CdtR phospho-variants in which our predicted phospho-accepting Asp, Asp61 was mutated for Ala or Glu. The constructs were assessed for their ability to restore CDT production. Dephospho-CdtR-Asp61Ala was completely non-functional and mirrored the *cdtR*-deletion mutant, whilst phospho-CdtR-Asp61Glu was functional, possessing 38–52% of wild-type activity. Taken together, these data suggest that CdtR is activated by phosphorylation of Asp61. The same principles were applied to assess the function of PCR ribotype 078-derived CdtR, which was shown to be non-functional owing to polymorphisms present within its coding gene. Conversely, polymorphisms present within its promoter region, provide significantly enhanced promoter activity compared with its PCR ribotype 027 counterpart. To ensure our data were representative for each ribotype, we determined that the *cdtR* nucleotide sequence was conserved in a small library of eight PCR ribotype 027 clinical isolates and nineteen PCR ribotype 078 isolates from clinical and animal origin.

## Highlights

• R20291ΔPaLocΔ*cdtR* model strains were applied to study the toxin regulator CdtR.
• (de)phosphomimetic substitutions revealed that CdtR is activated by phosphorylation of Asp61.
• Ribotype 078 CdtR was shown to be non-functional.
• P_*cdtR*_ derived from ribotype 078 has much stronger activity than its ribotype 027 counterpart.
• *cdtR* nucleotide sequence is conserved within eight ribotype 027 and nineteen ribotype 078 strains.

## 1 Introduction

*Clostridium difficile* (recently reclassified as *Clostridioides difficile* [1]), is the leading cause of hospital-associated diarrhoea in the developed world. In 2011, there were an estimated 453,000 cases and 29,000 deaths in the USA alone [2]. The main virulence factors of *C. difficile* are the monoglucosyltransferases, Toxin A (TcdA) and Toxin B (TcdB) [3].

Recently, the contribution of the *C. difficile* transferase (CDT), or the binary toxin, to disease pathogenesis, is becoming increasingly clear. The ADP-ribosylating toxin is comprised of an enzymatic sub-unit, CDTa which ADP-ribosylates monomeric actin thus preventing actin polymerisation causing cell rounding, and the formation of microtubule protrusions, and a binding sub-unit, CDTb, which permits cellular entry of CDTa [4]. In non-outbreak situations, 17–23% of clinical isolates possess CDT genes [5,6]. Purified [7], and supernatant-derived CDT [8], are toxic to mammalian cell-lines, whilst purified CDT is also lethal in rodent models of *C. difficile* infection (CDI) [9]. Isogenic mutants of R20291 producing only CDT, were shown to cause symptomatic CDI in hamsters, in parallel, the co-expression of CDT, increases the virulence of mutants producing either TcdA, TcdB or both [10,11]. Not only is CDT associated with the hypervirulent PCR ribotype (RT) 027, for example strain R20291 [12], but clinical cases of CDI attributed to TcdA^−^, TcdB^−^, CDT^+^ strains, have recently been described [13,14]. Collectively, these experimental and clinical data provide a strong argument for the contribution of CDT to the pathogenesis of *C. difficile*, thus substantiating the need for research into its genetic regulation.

Expression of *cdtA* and *cdtB* is linked to an upstream gene *cdtR*, encoding an orphan two-component signal transduction system (TCS) response regulator (RR), belonging to the LytTR family [8,15]. We recently developed R20291ΔPaLoc model strains devoid of TcdA/TcdB activity, for the study of CDT. Using these strains and *in vitro* cytotoxicity assays, we showed that CdtR was required for the production of CDT to cytotoxic levels towards Vero cell-lines, through the in-frame deletion and chromosomal complementation of *cdtR* [8]. CdtR also regulates the production of TcdA/TcdB in RT 027 [15]. Despite these observations, the TCS signal transduction pathway, leading to the activation of CdtR, remains uncharacterised.

RT 078 strains possess CDT genes but few studies have investigated CDT production in RT 078 strains, instead, the detection of *cdtA*/*cdtB* genes is usually described. Whilst a truncating substitution is present within *cdtR* [16], the functionality of the RT 078 CdtR homolog has not yet been determined.

In this study, we generated R20291ΔPaLocΔ*cdtR* model strains, expressing CdtR phospho-variants and RT 078-derived CdtR. Their application to our recently developed cytotoxicity assays identified the phosphorylation site at which CdtR is activated, and demonstrated a lack of function for RT 078 CdtR.

## 2 Materials and methods

### 2.1 Generation of strains expressing phospho-variant and RT 078-derived CdtR

Strains used in this study are listed in Table 1. Plasmids and primers are detailed in Tables S1 and S2, in the supplementary information. *cdtR* coupled with its 273bp promoter, was amplified by PCR using *cdtR*-promoter F/*cdtR*-6xhis R primers and cloned into pMTL-YN2C by means of flanking NotI and BamHI restriction sites. This plasmid is identical to pMTL-YN2C-*cdtR* [8] with the addition of a C-terminal hexahistidine tag (6xhis) to facilitate downstream purification. Thereafter, the potentially dephosphomimetic Asp61Ala (D61A) construct was generated by inverse PCR site-directed mutagenesis, with D61A SDM F/R primers, using the Q5 site-directed mutagenesis kit (NEB, USA) according to the manufacturer's instructions, using pMTL YN2C-*cdtR* 6xhis as a template. The potentially phosphomimetic Asp61Glu (D61E) construct was generated by site-directed mutagenesis by PCR and mutagenic splicing. To this end, two PCR reactions were conducted with *cdtR* promoter F/*cdtR* mut R primers, and *cdtR* mut F/*cdtR* 6xhis R primers, to form two amplicons, each containing a complementary 26bp mutagenic region encoding the Asp61Glu substitution. After which, the two fragments were spliced together using splicing by overlap extension (SOEing) PCR, with promoter *cdtR* F/*cdtR* 6xhis R primers. The ensuing fragment was cloned into pMTL-YN2C. RT 078-derived *cdtR* was amplified from strain M120 using *cdtR*-promoter F/M120-cdtR R primers and cloned into pMTL YN2C. The promoter-*cdtR* constructs for pMTL-YN2C-*cdtR*-6xhis, pMTL-YN2C-*cdtR*-D61A-6xhis, pMTL-YN2C-*cdtR*-D61E-6xhis and pMTL-YN2C-M120-cdtR, were confirmed to be as intended by Sanger sequencing. All four plasmids were then conjugated into the model strain R20291Δ*pyrE*ΔPaLocΔ*cdtR,* before the *cdtR* variants were knocked-in at the *pyrE* locus preceding plasmid loss, confirmed on the basis of thiamphenicol sensitivity, exactly as described previously [8]. Accordingly, uracil prototrophs were screened for *cdtR* insertions at *pyrE* using *pyrE* WT F/*cdtR* 6xhis R primers. The presence of approximately 1800bp products, demonstrate the correct insertions (Fig. S1), the extent of which was confirmed by Sanger sequencing.

**Table 1:** Strains used in this study.

Strain	Description	Reference/Origin
***E. coli***	Cloning host. Conjugation host	Invitrogen, USA (Williams et al., 1990)
Top10		
CA434		
***C. difficile***	Clinical RT 027 isolate	J. Brazier, Anaerobe Reference Laboratory, Cardiff, United Kingdom
R20291		
R20291Δ*pyrE*ΔPaLocΔ*cdtR*	Model strain for complementation	[8]
R20291ΔPaLoc	*pyrE-*restored mutant.	[8]
R20291ΔPaLocΔ*cdtR*	*pyrE-r*estored mutant.	[8]
R20291ΔPaLocΔcdtR*cdtR	*cdtR*-complemented mutant	[8]
R20291ΔPaLocΔcdtR*M120-cdtR	M120 cdtR complement	This study
R20291ΔPaLocΔcdtR*cdtR-his	6xhis *cdtR* complement	This study
R20291ΔPaLocΔcdtR*cdtR D61A-his	6xhis phospho-*cdtR* complement	This study
R20291ΔPaLocΔcdtR*D61E-his	6xhis dephospho-*cdtR* complement	This study
DH1916	Clinical RT 027 isolate	Val Hall
L2 (31,568)	Clinical RT 027 isolate	Ed Kujiper
L6 (5,108,111)	Clinical RT 027 isolate	Ed Kujiper
L8 (32,219)	Clinical RT 027 isolate	Ed Kujiper
L10 (2191)	Clinical RT 027 isolate	Ed Kujiper
L14 (60,902)	Clinical RT 027 isolate	Ed Kujiper
L16 (26,131)	Clinical RT 027 isolate	Ed Kujiper
M120	Clinical RT 027 isolate	[18]
Wilcox 078	Clinical RT 078 isolate	Mark Wilcox
EK23 (Type 078)	Clinical RT 078 isolate	Ed Kujiper
EK24 (CD2315)	Clinical RT 078 isolate	Ed Kujiper
EK26 (2016)	Clinical RT 078 isolate	Ed Kujiper
EK27 (7,004,578)	Clinical RT 078 isolate	Ed Kujiper
EK28 (7,009,825)	Clinical RT 078 isolate	Ed Kujiper
CL5499	Clinical RT 078 isolate	Christina Rodriguez
CL5502	Pig RT 078 isolate	Christina Rodriguez
CL5503	Pig RT 078 isolate	Christina Rodriguez
CL5504	Pig RT 078 isolate	Christina Rodriguez
CL5506	Pig RT 078 isolate	Christina Rodriguez
CL5655	Pig RT 078 isolate	Christina Rodriguez
CL5656	Pig RT 078 isolate	Christina Rodriguez
CL5657	Pig RT 078 isolate	Christina Rodriguez
CL5695	Pig RT 078 isolate	Christina Rodriguez
CL5696	Pig RT 078 isolate	Christina Rodriguez
CL5698	Pig RT 078 isolate	Christina Rodriguez
CL6136	Pig RT 078 isolate	Christina Rodriguez

### 2.2 Assessment of CDT-mediated virulence

CDT-mediated virulence was determined exactly as described previously through Western blot and cytotoxicity assays [8]. The production of CdtA was assessed qualitatively by Western blot. In brief, 24, 48 and 96h supernatants were filter-sterilized and the protein contents concentrated 40X with trichloracetic acid. Concentrated protein was then separated by SDS-PAGE and transferred to a PVDF membrane, which was blocked in a 5% (w/v) solution of skimmed milk, before overnight incubation with an HRP-Chicken anti-*Clostridium difficile* Binary Toxin Subunit A antibody (Gallus-Immunotech). Membranes were washed in tris-buffered saline containing 0.1% Tween 20, and developed with 3,3′,5,5′-Tetramethylbenzidine substrate solution (Sigma Aldrich). CDT-mediated virulence was determined from 24, 48 and 96h trypsin-activated, filter-sterilized supernatants. Supernatants were applied to 96 well-plates with each well containing a monolayer of approximately 1.5 × 10^3^ Vero cell-lines alongside the appropriate model strain supernatants and controls, and incubated for 24h. Representative images were taken for each treated monoculture and the cytotoxicity for each test strain was determined by the quantification of rounded cells therefrom, as a consequence of actin ADP-ribosylation. The cytotoxicity of each test strain was also expressed as a relative percentage to full cytotoxicity, imparted by the model strain R20291ΔPaLocΔ*cdtR* supernatant.

### 2.3 Assessment of R20291 and M120-derived P_*cdtR*_

The promoter regions of R20291 and M120 were amplified using *cdtR* promoter F/R primers and cloned upstream of the *catP* reporter gene in pMTL82254 [17], by means of flanking NotI and NdeI restriction sites. Plasmids harbouring R20291 and M120-derived P_*cdtR*_-*catP* fusions, as well as no-promoter control, were transformed into *E. coli* Top10 and maintained on the basis of erythromycin resistance. A 1 μL sterile loop of solid medium-derived culture, was harvested for each replicate of each strain, and sub-cultured into 5 mL Luria Bertani (LB) medium supplemented with 25 μg/mL chloramphenicol. The optical density (OD_600nm_) was measured for each replicate before incubating for 24 h at 37 °C with 200 rpm shaking, after which, repeat OD measurements were taken. Promoter activity was expressed as the fold-change in OD_600nm_ following overnight incubation in the presence of chloramphenicol.

### 2.4 Sequence analysis of RT 027 and RT 078 cdtR

*cdtR* was amplified from eight RT 027 strains and nineteen RT 078 strains (Table 1), using *cdtR*-promoter F/*cdtR*-6xhis R and *cdtR*-promoter F/M120-cdtR R primers respectively. The nucleotide sequences were subsequently confirmed by Sanger sequencing and aligned to the sequence of R20291 or M120-derived *cdtR*, using the molecular biology web tool Benchling.

## 3 Results and discussion

### 3.1 Prediction of the phospho-accepting Asp

Bacterial TCS are typically comprised of two proteins, a trans-membrane histidine kinase (HK) which receives an environmental stimulus preceding autophsophorylation, and a DNA-binding RR to which the phosphoryl group is transferred [19]. The HK(s) with which CdtR interacts are yet to be identified, whilst the amino acid residue at which CdtR is phosphorylated, has not been determined. Fundamental to TCS signal transduction is phosphoransfer from HK to RR. RRs are comprised of two distinct domains, a conserved N-terminal receiver domain (REC), belonging to the REC superfamily (pfam00072), and a variable C-terminal DNA-binding effector domain, or output domain. Phosphorylation occurs within the REC domain of RRs at a conserved Asp, for example Asp 54 of NtrC type RRs [20], after which, conformational change activates the proteins permitting target gene regulation often through phosphorylation-mediated homodimerization, preceding promotor binding by the output domain [21]. CdtR contains seven Asp residues within its predicted REC domain: Asp 2, Asp 7, Asp 9, Asp 30, Asp 32, Asp61 and Asp 72.

In order to identify potential phospho-accepting Asp residues within CdtR, the amino acid sequence of the R20291 REC domain, was aligned with the top 10 listed sequences of the REC superfamily (pfam00072), using the NCBI conserved domain search (CDS) server. Asp61 was completely conserved amongst all 10 members, which was located in a string of four amino acids comprising a non-polar hydrophobic residue, followed by a non-conserved residue, a non-polar hydrophobic residue and finally, Asp61 (Fig. S2). The strong conservation of Asp61 led us to hypothesise this residue as the phospho-acceptor for CdtR.

### 3.2 CdtR is activated by phosphorylation of Asp61

**Fig. 1:**
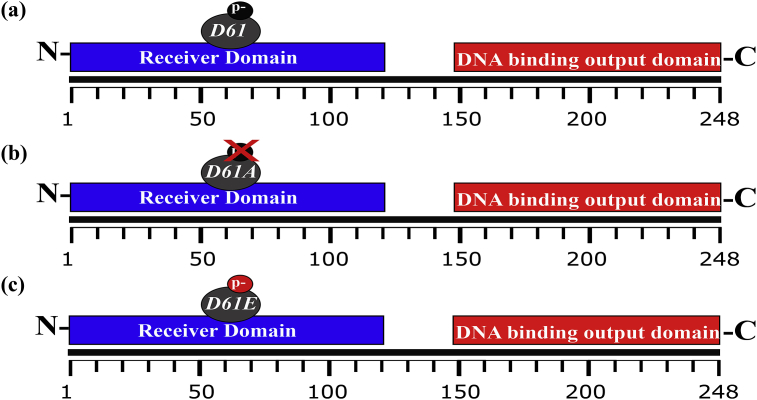
Hypothesised effects of CdtR phospho-variant substitutions a) wild-type CdtR is phosphorylated at Asp61 and is consequently functional b) Asp61Ala (D61A) is dephosphomimetic and is consequently non-functional c) Asp61Glu (D61E) is potentially phosphomimetic and may possess activity independent from phosphorylation by its partnered TCS HK(s). Single column.

We next investigated the effects of (de)phosphomimetic substitutions of Asp61 upon the function of CdtR. Substitution of the phosphoryl-accepting Asp with Ala renders a RR inactive owing to its inability to receive a phosphoryl group from its partner HK. Conversely, substitution of the phosphoryl-accepting Asp with Glu can be phosphomimetic and render the RR active without the requirement for phosphorylation, through extension of the distance between the negatively charged carboxyl group on the amino acid side-chain and the α-carbon on the amino acid backbone [22,23]. We hypothesised that wild-type CdtR is functional owing to its phosphorylation at Asp61 (Fig. 1a) and that an Asp61Ala mutation would be non-functional owing to the removal of the phospho-accepting Asp (Fig. 1b). Moreover, if CdtR is a suitable candidate for phosphomimetic studies, then an Asp61Glu substitution might provide constitutive activity (Fig. 1c).

**Fig. 2:**
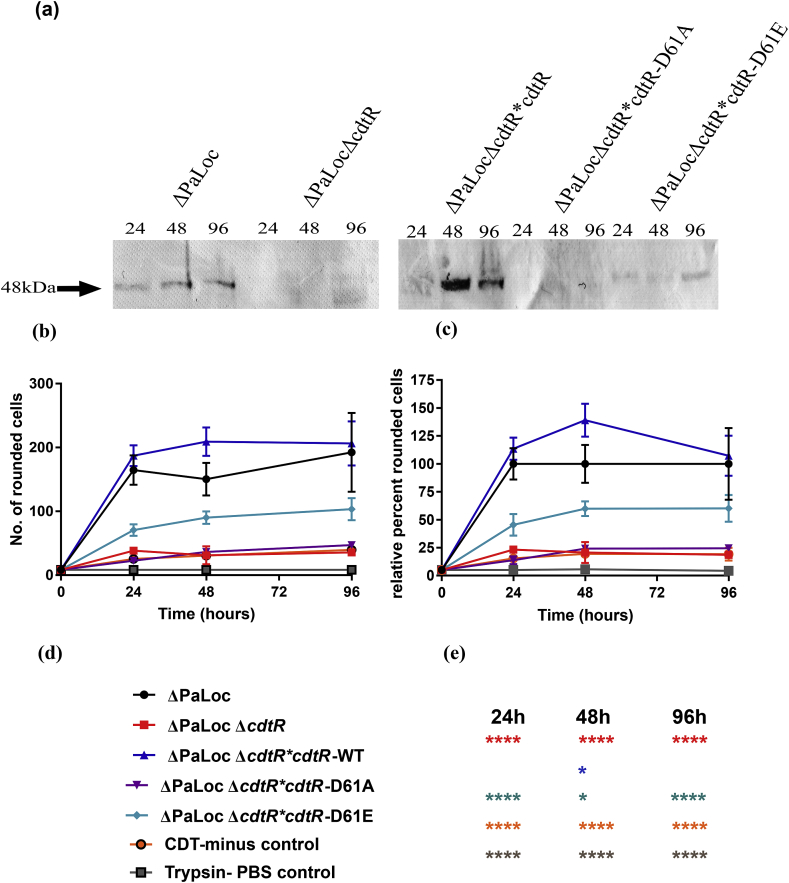
Effect of (de)phosphomimetic substitution on CDT-mediated virulence a) Western blot detection of CdtA from 24, 48 and 96h supernatants detected with an anti-CdtA:HRP antibody derived from strains R20291ΔPaLoc, R20291ΔPaLocΔ*cdtR*, R20291 ΔPaLocΔ*cdtR* *cdtR, R20291 ΔPaLocΔ*cdtR* *cdtR Asp61Ala (D61A) and R20291 ΔPaLocΔ*cdtR* *cdtR Asp61Glu (D61E) b) Total number of rounded cells c) Percentage of rounded cells relative to complete virulence by strain R20291ΔPaLoc, for Vero cells treated with the model strain-derived supernatants and relative controls. Data represent the mean ± SD of three replicate values except for D61E replicate 3 and CDT-minus control replicate 1 whose images at the 48h time point were duplicates of each other d) key of strains and controls e) statistical significance compared to the ΔPaLoc parental according to Two-Way ANOVA followed by Tukey's multiple comparison test P = *<0.05, ***<0.001, ****<0.0001. Double column.

The model strain R20291ΔPaLocΔ*cdtR* was chromosomally complemented with constructs encoding CdtR-6xhis as well as the two putative CdtR phospho-variants. The mutants were then assessed for their ability to restore CDT production through a combination of Western blot and cytotoxicity analysis. Three time points were chosen for study, to ascertain whether a phosphomimic could induce CDT production earlier than normally observed. Our results mimicked those seen in our previous article [8], in which the ΔPaLoc parental strain produced detectable CDTa across all three time points, whilst none was detectable for the ΔPaLocΔ*cdtR* supernatant (Fig. 2a). This production was restored by the integration of *cdtR*-6xhis at the *pyrE* locus, thus indicating that the tag does not alter protein function. Chromosomal complementation of *cdtR* led to an overproduction of CDTa compared with the parental strain. This observation was detailed in our previous publication in which the complemented *cdtR* mutant posessed ≥4-fold more CDT activity as assessed by cytotoxicity assay [8]. This is most likely ascribed to the design of the pMTL-YN2C complementation vector, which does not possess a transcriptional terminator positioned between the end of *pyrE* and the start of the MCS for gene complementation. No CDTa was detected for the Asp61Ala complement which was comparable to the ΔPaLocΔ*cdtR* parental, thereby validating the lack of function for this phospho-null CdtR variant, hereafter referred to as dephospho-CdtR (Fig. 2a). Finally, CDTa was detectable in the ΔPaLocΔ*cdtR* *cdtR Asp61Glu (D61E) supernatants, although substantial detection was not seen until the 96h time-point signifying only partial activity (Fig. 2a). Restoration of the *cdtR*-null phenotype by the Asp61Glu phospho-mimic, hereafter referred to phospho-CdtR, consolidates the notion that CdtR is activated by phosphorylation of Asp61. In order to substantiate this observation with the physical differentiation of phospho-CdtR and dephospho-CdtR, attempts were made to purify the CdtR-6xhis fusions directly from *C. difficile* lysates by means of their C-terminal 6xhis tags. Unfortunately, we were unable to obtain the level of CdtR required for positive detection by Western blot using an anti-6xhis antibody. Moreover, the orphan nature of CdtR prevents *in vitro* phosphorylation assays, since the activating HK has not yet been identified.

We next assessed the effects of expressing CdtR phospho-variants on the relative CDT-mediated cytotoxicity of each strain using our cytotoxicity assay. Supernatants derived from the ΔPaLoc parental strain rounded an average of 165, 150 and 192 cells at the 24, 48 and 96h time points (Figs. S3–S5 a-c), representing the 100% virulence benchmark (Fig. 2b–c). Application of the ΔPaLocΔ*cdtR* supernatant rounded 38, 31 and 40 cells across the three time points (Figs. S3–S5 d-f) representing 23, 21 and 19% cytotoxicity compared with the ΔPaLoc parental strain. These values were similar to those obtained following treatment with the CDT-minus control, in which CDTb present in the ΔPaLoc supernatant had not been proteolytically activated with trypsin and consequently could not enter the cells through receptor-mediated endocytosis [24,25]. Such treatments led to 25, 31 and 40 (Figs. S3–S5 p-r) rounded cells representing 15, 19 and 19% cytotoxicity relative to the trypsinised ΔPaLoc supernatants (Fig. 2b–c), thus supporting our previous observation, that within our experimental system, CdtR is required for the production of CDT to cytotoxic levels. CDT-mediated cytotoxicity, was restored by complementation with CdtR-6xhis at the *pyrE* locus. Treatment with these supernatants rounded an average of 107, 209 and 206 cells across the three time-points representing 114, 139 and 107% cytotoxicity (Figs. S3–S5 g-i), relative to the ΔPaLoc parental. Complementation with CdtR-Asp61Ala led to 23, 36 and 47 rounded cells across the three time points (Figs. S3–S5 j-l), which represented 14, 24 and 24% percent relative cytotoxicity (Fig. 2b–c), thus validating the lack of function for dephospho- CdtR, since these values closely resemble those of the Δ*cdtR* strain. Conversely, complementation with phospho-*cdtR* led to a partial restoration of CDT-mediated virulence. Across the three time points, this complement rounded an average of 71, 90 and 103 cells (Figs. S3–S5 m-o), providing 46, 60 and 60% of the relative cytotoxic effect compared with the ΔPaLoc parental, thereby substantiating the activity of the Asp61Glu phospho-mimic thus validating this residue as the phospho-accepting Asp. Owing to the increased production of CDT when *cdtR* is complemented at the *pyrE* locus, the values provided above likely overestimate the activity of phospho-CdtR when compared to the ΔPaLoc parental strain. Comparison with the 6xhis complement for a more accurate approximation, revealed a relative activity of 38, 43 and 52% across the 24, 48 and 96h time points respectively. The observation that CdtR is activated by phosphorylation of Asp61 uncovers a fundamental process in the TCS pathway, leading to the regulation of toxin production by CdtR.

### 3.3 RT 078 CdtR is non-functional

**Fig. 3:**
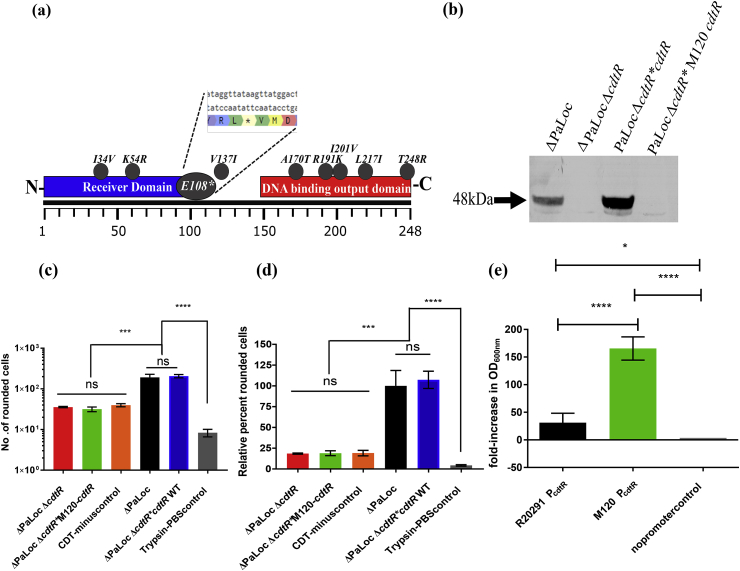
Effect of M120-cdtR complementation on CDT-mediated virulence a) Non-synonymous polymorphisms present within M120-CdtR compared with R20291 b) Western blot detection of CDTa from 96h supernatants detected with an anti-CdtA:HRP antibody derived from strains R20291ΔPaLoc, R20291ΔPaLocΔ*cdtR*, R20291 ΔPaLocΔ*cdtR* *cdtR, and R20291 ΔPaLocΔ*cdtR* *M120-cdtR c) Total number of rounded cells d) Percentage of rounded cells relative to complete virulence by strain R20291ΔPaLoc, for Vero cells treated with 96h model strain-derived supernatants and relative controls. Data represent the mean ± SD of 3 replicate values. e) R20291 and M120 P_cdtR_ promotor assay displaying the change in optical density values derived by promoter-driven transcription of *catP* following overnight incubation in the presence of chloramphenicol. Data represent the mean ± SD of 5 replicate values P = *<0.05, ***<0.001, ****<0.0001 according to One-Way ANOVA followed by Tukey's multiple comparison test. Double column.

Strain M120 is the archetypal RT 078 strain since it was the first to have its genome sequenced [26]. M120 possesses nine non-synonymous polymorphisms in *cdtR* compared with the sequence of R20291 (Fig. 3a), notably the truncating stop codon ensuing Glu108Stop. It is presumed that such a truncation would render M120-CdtR non-functional, however, this has never been experimentally verified. Moreover, an ATG start codon is located one trinucleotide after the truncation, although an obvious ribosomal binding site is not present in the immediate upstream region (Fig. 3a). We sought to test the function of M120-derived CdtR.

In a similar fashion to the approach described above, M120-cdtR was knocked in at the *pyrE* locus of R20291ΔPaLocΔ*cdtR* and assessed for its ability to restore CDT production. Following Western blot analysis, results looked similar to those described above, wherein CDTa production was clearly ablated following deletion of *cdtR,* which was subsequently restored following complementation at the *pyrE* locus (Fig. 3b). Complementation with M120-derived *cdtR* was unable to restore CdtA production therefore indicating a lack of function for this homolog, owing presumably to the truncating Glu108Stop polymorphism.

We next tested the relative CDT-mediated cytotoxicity of the strain expressing M120-CdtR. The cytotoxicity of this strain was measured at the 96h time-point alongside the assays described above. The M120-cdtR knock-in strain rounded an average of 32 cells (Fig. S5 v-x), compared with 192 of the ΔPaLoc parental (Fig. 3c). This relative cytotoxicity score of 19% is identical to that of the ΔPaLocΔ*cdtR* strain and the CDT-minus control (Fig. 3d) therefore substantiating the lack of function for this homolog.

Not only does the M120 cdtR ORF possess nine non-synonymous substitutions compared with R20291, but the upstream region containing the promoter also contains mutations compared with its R20291 counterpart. One single nucleotide polymorphism is present compared with R20291, whilst each promoter has one missing nucleotide from the P_*cdtR*_ alignment, resulting in three discrepancies between the two sequences. To ensure that the lack of functionality we have demonstrated for M120-CdtR stems from the ORF polymorphisms, and not those present within the promoter region, we assessed the function of R20291 and M120-derived P_*cdtR*_. The promoter regions were amplified from both strains and cloned into the reporter plasmid pMTL82254 [17]. *E. coli* TOP 10 harbouring each plasmid along with the empty promoter-null vector, were grown overnight in liquid LB containing chloramphenicol and assessed for their ability to withstand the antibiotic. The strain harbouring R20291-P_*cdtR*_*-catP* was clearly able to tolerate chloramphenicol, yielding a 31-fold increase in optical density following overnight incubation at 37 °C (Fig. 3e). The strain harbouring M120- P_*cdtR*_*-catP* was also resistant to chloramphenicol, in fact, the increase in OD_600nm_ was far greater than that observed for the R20291 construct with an increase by 166-fold. Finally, for the promoter-null empty vector, we observed a decrease in the optical density value by 0.11-fold relative to the starting value (Fig. 3e). Collectively, these data demonstrate that both R20291 and M120-derived *cdtR* promoter regions are functional and provide constitutive expression, therefore, the observed lack of function for M120-CdtR is surely a result of the non-synonymous substitutions within the ORF, presumably, the premature truncation. Further experimentation using alternative methods of promotor analysis would be required to precisely quantify the difference in promotor activity between RT 027 and RT 078-derived P_*cdtR*_

The contribution of CDT to the virulence of RT 078 remains to be determined and is beyond the remit of this article. In an early study, production of CDT by a representative RT 078 isolate was undetectable by Western blot [27]. In contrast, other studies have demonstrated the presence of *cdtA* transcripts [28], and secreted CDTa for RT 078 strains [15]. However, such production could be a consequence of polycistronic *cdtRAB* transcription which occurs from P_*cdtR*_ [29]. The observation that RT 078 CdtR is non-functional, suggests that this lineage may have evolved to overcome the requirement for CdtR-mediated regulation of CDT production, through increased activity of its polymorphic promoter, P_*cdtR*._ However, further experimentation would be required to determine this.

### 3.4 *cdtR* sequence is conserved within RT 027 and RT 078

The work presented here, demonstrates the functionality of R20291 and lack of functional for M120-derrived CdtR. However, whilst these archetypal strains are well studied, we needed to ascertain whether our data are representative for RT 027 and RT 078. To do this, we amplified and sequenced *cdtR* from seven RT 027 clinical isolates present in our SBRC culture collection, before aligning those sequences to that of R20291. In parallel, we amplified the same region from eighteen RT 078 isolates from clinical and animal origin and aligned these to M120. We observed complete nucleotide conservation within ribotypes, with a deviation of 4.4% between ribotypes (Table S3). The evidence stemming from this small dataset indicates that our data on CdtR functionality is likely representative for both RT 027 and RT 078. To our knowledge, all reported RT 078 *cdtR* sequences possess the truncating substitution with one exception. Strain CD98, listed as RT 078 in the respective study, was proposed to possess full-length *cdtR* encoding Glu at position 108 [28].

## 4 Conclusions

The expression of (de)phosphomimetic CdtR variants in R20291ΔPaLocΔ*cdtR* model strains allowed us to uncover a fundamental process in the TCS pathway leading to the regulation of toxin production by CdtR. Their application demonstrated that CdtR is activated by phosphorylation of Asp61. Meanwhile, expression of RT 078-derived CdtR, demonstrated its lack of function, owing to polymorphisms within its coding gene. Conversely, its polymorphic promoter region was considerably stronger than its RT 027 counterpart which potentially indicates a mechanism of evolution to overcome the requirement for CdtR-mediated regulation of CDT, through the acquisition of promoter polymorphisms. The nucleotide sequence of *cdtR* is conserved within RT 027 and RT 078 in a small library of clinical and animal isolates. Accordingly, these data should be representative for both RTs.

## Conflicts of interest

The authors declare no conflicts of interest.
