# Supplementary material for: Phosphorylation and functionality of CdtR in Clostridium difficile
Source: Anaerobe. 2019 Aug;58:103–9. doi: 10.1016/j.anaerobe.2019.102074 (PMC6699598; doi:10.1016/j.anaerobe.2019.102074)
Supplement: Multimedia component 1 [file mmc1.docx]

**Supplementary information**

**Phosphorylation and functionality of CdtR in *Clostridium difficile.***

T.W. Bilverstone^1^, N.P Minton^1,2^ and S.A. Kuehne^3^.

^1^ Clostridia Research Group, BBSRC/EPSRC Synthetic Biology Research Centre (SBRC), School of Life Sciences, Centre for Biomolecular Sciences, The University of Nottingham, Nottingham, NG7 2RD, UK.

^2^ NIHR Nottingham Biomedical Research Centre, Nottingham University Hospitals NHS Trust and the University of Nottingham, Nottingham, NG7 2RD, UK.

^3^Oral Microbiology Group, School of Dentistry, College of Medical and Dental Sciences, The University of Birmingham, Birmingham, B5 7EG, UK

Corresponding author: nigel.minton@nottingham.ac.uk

**Keywords:** *C. difficile*, CDT, CdtR, binary toxin, virulence, phosphorylation

**Table S1: Plasmids used in this study**

| Plasmid | Description | Reference/Origin | |
| --- | --- | --- | --- |
| pMTL-YN2C  pMTL-YN2C-*cdtR*-6xhis  pMTL-YN2C-*cdtR*-D61A-6xhis  pMTL-YN2C-*cdtR*-D61E-6xhis  pMTL-YN2C-M120-*cdtR*  pMTL-82254  pMTL-82254 R20291 P*_cdtR_*  pMTL-82254 M120 P*_cdtR_* | Complementation vector for R20291  Complementation of 6xhis-tagged *cdtR* insert Complementation of 6xhis-*cdtR* with phosphomimetic amino acid substitution  Complementation of 6xhis-*cdtR* with dephosphomimetic amino acid substitution  Complementation of  M120-derived *cdtR*  *catP* reporter plasmid  R20291 P_cdtR_-*catP* reporter fusion  M120 P_cdtR_-*catP* reporter fusion | | [[1](#_ENREF_1)]  This study  This study  This study  This study  [[2](#_ENREF_2)]  This study  This study |

**Table S2: Primers used in this study**

| Primer designation | Sequence 5’-3’ | Application |
| --- | --- | --- |
| *cdtR*-promoter F  *cdtR*-6xhis R  M120-*cdtR* R  D61A SDM F  D61A SDM R | TTTTTGCGGCCGCCACGTATAAGAATAAAAATTCCAG  TTTTTGGATCCTTAATGATGATGATGATGATGTGCTGTTTTAATAATGTTCTTTAAAATATTTC  TTTTTGGATCCTTATCTTTTAATAATGTTCTTTAAAATATTTC  AGCAAAATACAAGATAAGTAATGAATTATGTG  GCTACAACTATAAAATATATTTTTACTTTATTTTTTTCAG | *cdtR* cloning  *cdtR* cloning  M120 *cdtR* cloning  Site-directed mutagenesis  Site-directed mutagenesis |
| D61E mut F  D61E mut R  *cdtR*-promoter R  *pyrE* WT F | GTTGTAGAAGCAAAATACAAGATAAG  CTTATCTTGTATTTTGCTTCTACAAC  TTTTTCATATGTAAATACCCTCCTATAAAAAATTC  GGAGCTACTTGTATCCAAG | Site-directed mutagenesis  Site-directed mutagenesis  Promoter amplification  Confirmation of *pyrE* integration |

**Table S3: *cdtR* nucleotide conservation within a small library of RT 027 and RT 078 strains**

| **Strain** | **Ribotype** | **Homology to R20291 *cdtR*** | **Homology to M120**  ***cdtR*** |
| --- | --- | --- | --- |
| **R20291** | 027 | 100% | 95.6% |
| **DH1916** | 027 | 100% | 95.6% |
| **L2** | 027 | 100% | 95.6% |
| **L6** | 027 | 100% | 95.6% |
| **L8** | 027 | 100% | 95.6% |
| **L10** | 027 | 100% | 95.6% |
| **L14** | 027 | 100% | 95.6% |
| **L16** | 027 | 100% | 95.6% |
| **M120** | 078 | 95.6% | 100% |
| **Wilcox 078** | 078 | 95.6% | 100% |
| **EK24** | 078 | 95.6% | 100% |
| **EK26** | 078 | 95.6% | 100% |
| **EK27** | 078 | 95.6% | 100% |
| **EK28** | 078 | 95.6% | 100% |
| **CL5499** | 078 | 95.6% | 100% |
| **CL5502** | 078 | 95.6% | 100% |
| **CL5503** | 078 | 95.6% | 100% |
| **CL5504** | 078 | 95.6% | 100% |
| **CL5506** | 078 | 95.6% | 100% |
| **CL5655** | 078 | 95.6% | 100% |
| **CL5656** | 078 | 95.6% | 100% |
| **CL5657** | 078 | 95.6% | 100% |
| **CL5695**  **CL5696**  **CL5698**  **CL6136** | 078  078  078  078 | 95.6%  95.6%  95.6%  95.6% | 100%  100%  100%  100% |


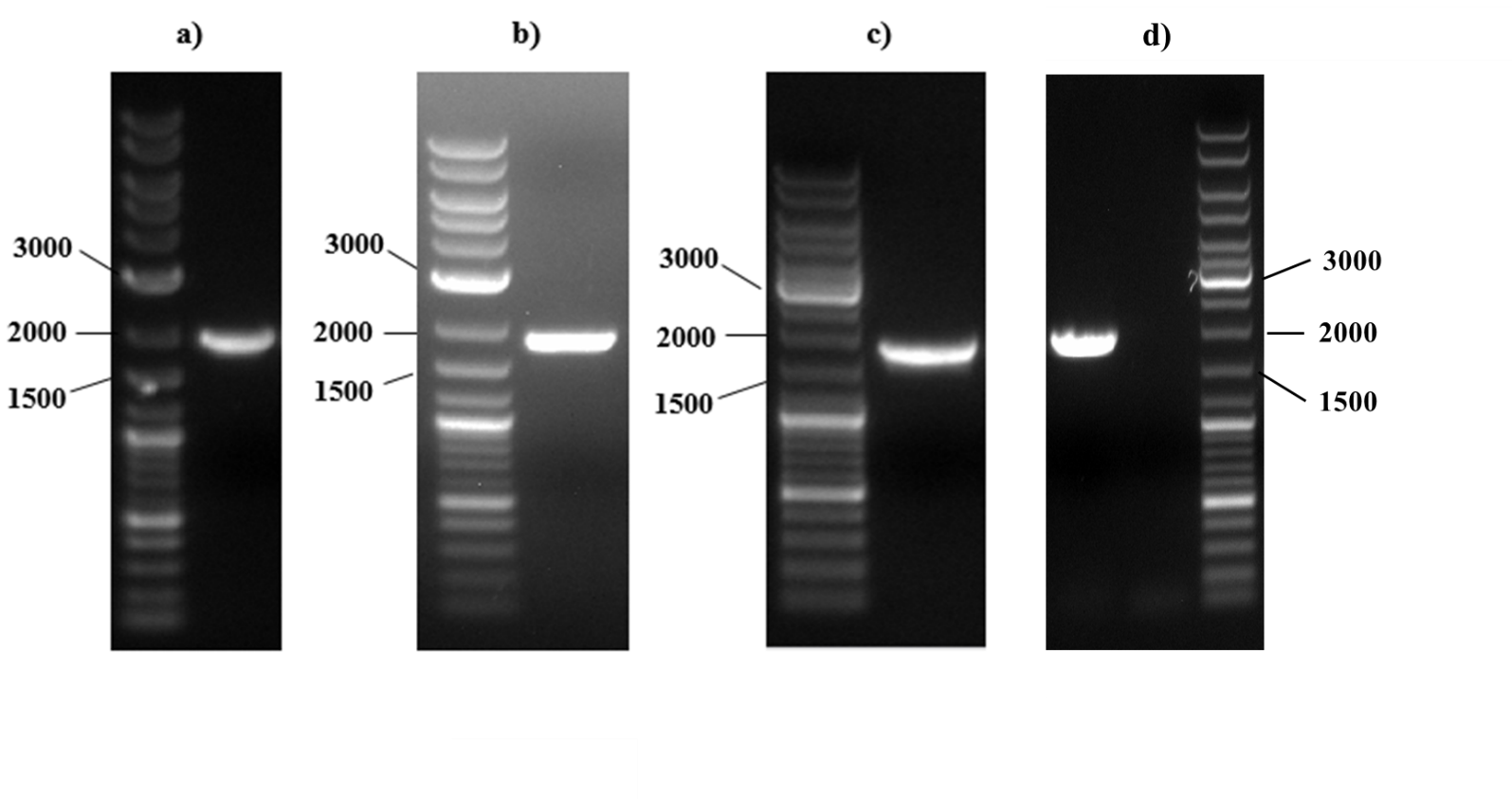


**Fig S1: Authentication of variant *cdtR* complementation at the *pyrE* locus.**

Gel images following PCR of **a)** *pyrE-cdtR*-6xhis beside an NEB 2-log ladder; **b)** *pyrE-cdtR*-D61A-6xhis beside an NEB 2-log ladder; **c)** *pyrE-cdtR*-D61E-6xhis beside a Thermo gene ruler ladder mix; **d)** *pyrE*-M120-*cdtR* beside negative control and a Thermo gene ruler ladder mix.


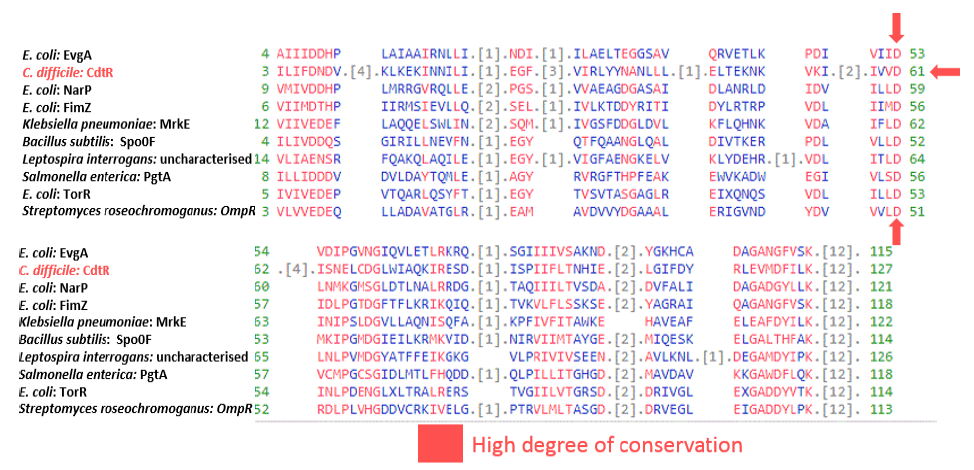


Figure S2: CDS alignment of the R20291 CdtR REC domain with top 10 listed sequences of the pfam00072 REC superfamily. Asp61 is indicated with red arrows.

**
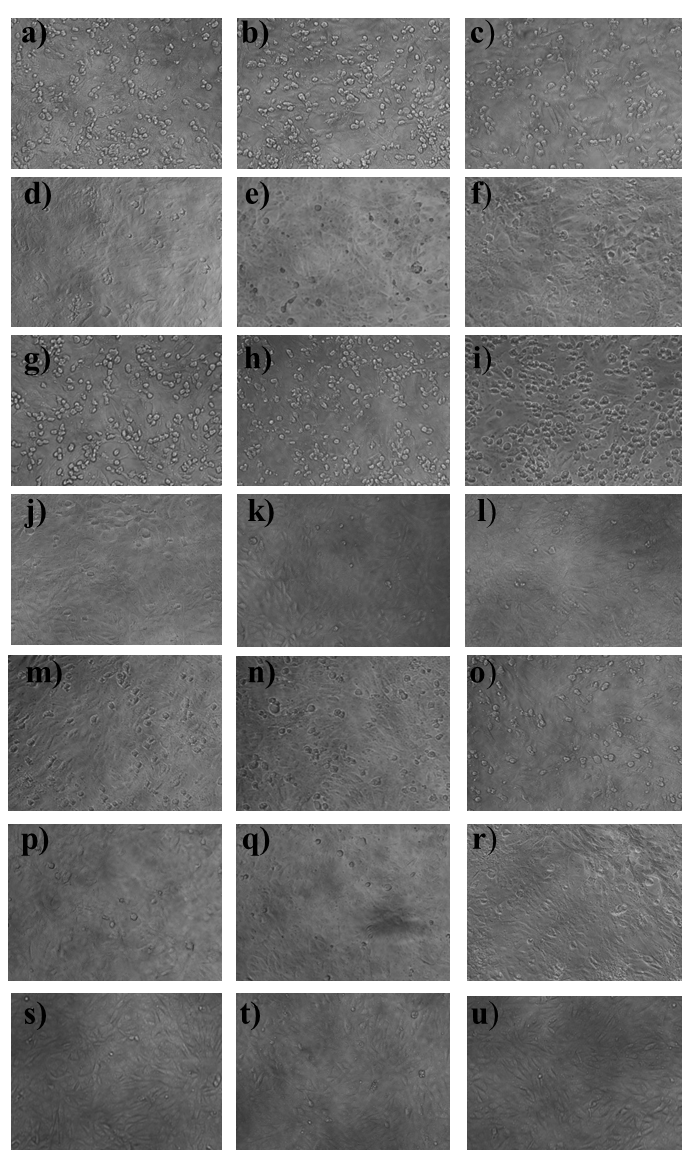
Figure S3: 24h Vero cell-cytotoxicity images for phosho-variant CdtR.** Representative images of Vero cells treated with 24h model strain derived supernatants and appropriate controls, (a-c): R20291∆PaLoc; (d-f): R20291∆PaLoc∆*cdtR*; (g-i): R20291∆PaLoc∆*cdtR***cdtR*; (j-l) R20291∆PaLoc∆*cdtR***cdtR-*Asp61Ala (D61A)*;* (m-o): R20291∆PaLoc∆*cdtR***cdtR-*Asp61Glu (D61E); (p-r): R20291∆PaLoc CDT-minus control*;* (s-u): Trypsin-PBS control.


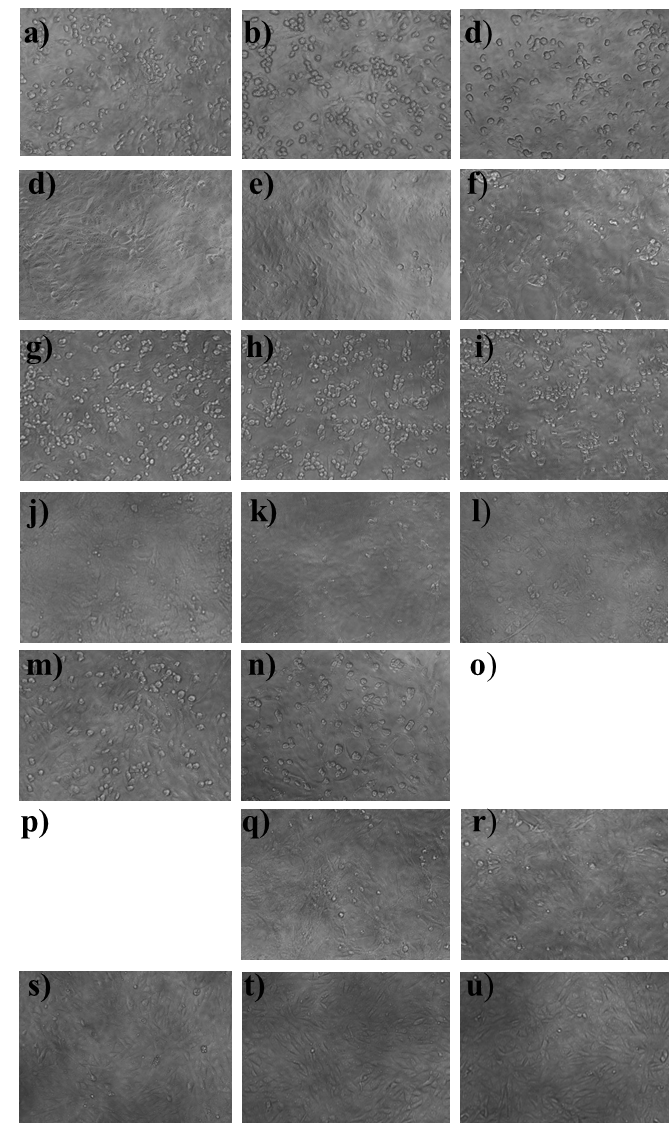


**Figure S4: 48h Vero cell-cytotoxicity images for phosho-variant CdtR.** Representative images of Vero cells treated with 96h model strain derived supernatants and appropriate controls, (a-c): R20291∆PaLoc; (d-f): R20291∆PaLoc∆*cdtR*; (g-i): R20291∆PaLoc∆*cdtR***cdtR*; (j-l) R20291∆PaLoc∆*cdtR***cdtR-*Asp61Ala (D61A)*;* (m-o): R20291∆PaLoc∆*cdtR***cdtR-*Asp61Glu (D61E); (p-r): R20291∆PaLoc CDT-minus control*;* (s-u): Trypsin-PBS control.


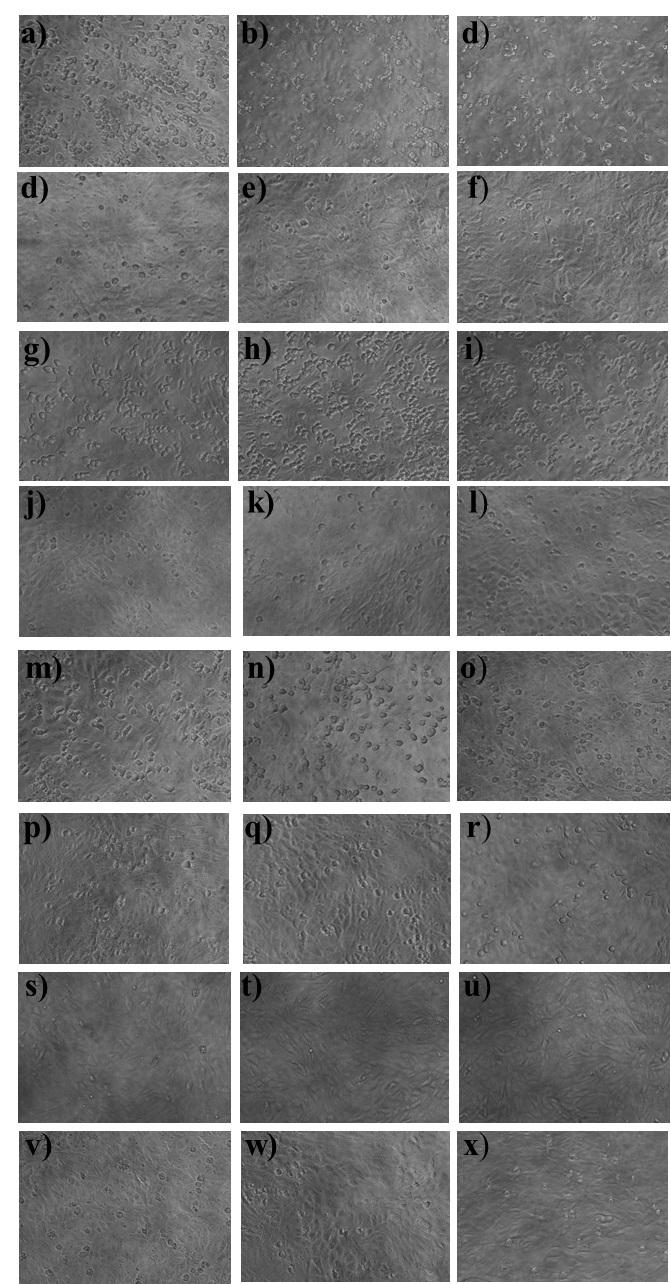
**Figure S5: 96h Vero cell-cytotoxicity images for phosho-variant CdtR.** Representative images of Vero cells treated with 96h model strain derived supernatants and appropriate controls, (a-c): R20291∆PaLoc; (d-f): R20291∆PaLoc∆*cdtR*; (g-i): R20291∆PaLoc∆*cdtR***cdtR*; (j-l) R20291∆PaLoc∆*cdtR***cdtR-*Asp61Ala (D61A) (m-o): R20291∆PaLoc∆*cdtR***cdtR*-Asp61Glu (D61E);; (p-r): R20291∆PaLoc CDT-minus control*;* (s-u): Trypsin-PBS control; (v-x): R20291∆PaLoc∆*cdtR** M120-*cdtR.*

**References**

1. Ng, Y.K., M. Ehsaan, S. Philip, M.M. Collery, C. Janoir, A. Collignon, S.T. Cartman, and N.P. Minton, Expanding the Repertoire of Gene Tools for Precise Manipulation of the *Clostridium difficile*Genome: Allelic Exchange Using *pyrE* Alleles*.* *PLoS ONE*, 2013. **8**(2): p. e56051.DOI: 10.1371/journal.pone.0056051.

2. Heap, J.T., O.J. Pennington, S.T. Cartman, and N.P. Minton, A modular system for Clostridium shuttle plasmids*.* *J Microbiol Methods*, 2009. **78**(1): p. 79-85.DOI: 10.1016/j.mimet.2009.05.004.
